# Genomic Characterization of a Novel Gut Symbiont From the Hadal Snailfish

**DOI:** 10.3389/fmicb.2019.02978

**Published:** 2020-01-10

**Authors:** Chun-Ang Lian, Guo-Yong Yan, Jiao-Mei Huang, Antoine Danchin, Yong Wang, Li-Sheng He

**Affiliations:** ^1^Institute of Deep-Sea Science and Engineering, Chinese Academy of Sciences, Sanya, China; ^2^College of Earth Sciences, University of Chinese Academy of Sciences, Beijing, China; ^3^Department of Infection, Immunity and Inflammation, Institut Cochin, INSERM U1016 – CNRS UMR 8104 – Université Paris Descartes, Paris, France

**Keywords:** hadal symbiosis, Tenericutes, CRISPR, snailfish, metagenome

## Abstract

Hadal trenches are characterized by not only high hydrostatic pressure but also scarcity of nutrients and high diversity of viruses. Snailfishes, as the dominant vertebrates, play an important role in hadal ecology. Although studies have suggested possible reasons for the tolerance of hadal snailfish to high hydrostatic pressure, little is known about the strategies employed by hadal snailfish to cope with low-nutrient and virus-rich conditions. In this study, the gut microbiota of hadal snailfish was investigated. A novel bacterium named “*Candidatus* Mycoplasma liparidae” was dominant in the guts of three snailfish individuals from both the Mariana and Yap trenches. A draft genome of “*Ca.* Mycoplasma liparidae” was successfully assembled with 97.8% completeness by hybrid sequencing. A set of genes encoding riboflavin biosynthesis proteins and a clustered regularly interspaced short palindromic repeats (CRISPR) system was present in the genome of “*Ca.* Mycoplasma liparidae,” which was unusual for *Mycoplasma*. The functional repertoire of the “*Ca.* Mycoplasma liparidae” genome is likely set to help the host in riboflavin supplementation and to provide protection against viruses via a super CRISPR system. Remarkably, genes encoding common virulence factors usually exist in Tenericutes pathogens but were lacking in the genome of “*Ca.* Mycoplasma liparidae.” All of these characteristics supported an essential role of “*Ca.* Mycoplasma liparidae” in snailfish living in the hadal zone. Our findings provide further insights into symbiotic associations in the hadal biosphere.

## Introduction

The hadal zone, at water depths below 6,000 m, comprises mainly trenches that plunge from the abyssal plains to depths of up to 11,000 m and is the least explored region on Earth (Somero, 1992; Jamieson et al., 2010). In contrast to the upper abyssal oceans, the trench environment represents a distinct and often extreme deep habitat with some striking features. The primary characteristic of the hadal zone is high hydrostatic pressure, which increases with depth, reaching up to 1.1 tons per square centimeter in the deepest ocean trenches (Jamieson et al., 2010; Nunoura et al., 2015). Moreover, the light intensity in the hadal zone is too low to sustain photosynthetic primary production, and the availability of organic matter that is mainly supplemented by surface-derived carrion falls controls benthic biomass (Jorgensen and Boetius, 2007). However, most of the sinking material is consumed and intercepted by plankton and heterotrophic bacteria in shallower and bathyal waters that selectively remove highly labile compounds before the material reaches the trenches. Thus, trench communities are typically considered nutrition-limited systems (Jamieson et al., 2010). In addition, deep-sea ecosystems are rich with viruses, which are a predominant cause of the mortality of microorganisms, which in turn contribute to biogeochemical cycles (Hara et al., 1996; Yoshida et al., 2013; Anantharaman et al., 2014). In deep-sea environments, prokaryotic mortality due to viruses is estimated to be dozens of times more than that due to other causes (Lara et al., 2017). Although the hadal environment is extreme, diverse organisms inhabit this zone. How these creatures survive in such extreme conditions is still unclear. Sampling difficulties are the main reasons, and studies on these topics have progressed very slowly. Fishes are the only vertebrates inhabiting the hadal zone, and snailfishes are the dominant ichthyofauna at depths of approximately 6,000–8,145 m (Stein, 2005; Linley et al., 2016). A previous study showed that the osmolyte trimethylamine *N*-oxide (TMAO) helped snailfish stabilize proteins against pressure (Yancey et al., 2014). With the interpretation of the hadal snailfish genome, the mechanism underlying the adaptation of hadal snailfish to pressure has been understood from a new perspective (Wang et al., 2019). However, with regard to the characteristics of the hadal environment, in addition to pressure, nutrient acquisition, micronutrient biosynthesis and immune protection are also critical for organismal survival due to the low nutrient levels under normal conditions and the high abundance of unknown viruses and other potentially harmful microorganisms in the surrounding water compared to the surface (Hara et al., 1996; Yoshida et al., 2013).

The gut is a very important organ for the survival and health of organisms in many respects. Almost all of these biological activities are accomplished with the assistance of intestinal microbes (Ley et al., 2008; Nicholson et al., 2012; Douglas, 2015; Paniagua Voirol et al., 2018). This phenomenon is particularly evident for organisms in deep sea. In the stomach microbiota of *Bathynomus* sp., a deep-sea isopod living at a depth of 898 m in the South China Sea, symbiotic mycoplasmas occupy 42.8–100% of the microbial communities and might provide sugars and amino acids to the hosts (Wang et al., 2016). A sea cucumber from a depth of > 6,000 m in the Mariana Trench harbored obligate symbiotic spiroplasmas, with 63.5% occupation in the hindgut bacterial community. *Spiroplasma* symbionts might be able to protect the host against viral infections through clustered regularly interspaced short palindromic repeats (CRISPR) systems (He et al., 2017). For the hadal dominant species *Hirondellea gigas*, Tenericutes were also found in the gut microbial communities in the Mariana Trench (Zhang et al., 2018). Distinct from these deep-sea scavengers, the hadal snailfish primarily feeds on amphipods (Gerringer et al., 2017b), serving as a major player in deep-sea ecosystems and on the top of the food chain. The microbial inhabitants in the snailfish gut and their contributions to host survival remain unknown. In this study, we will characterize a novel mycoplasma, the dominant microbe in the hadal snailfish gut, and describe its contributions to hadal snailfish survival.

## Materials and Methods

### Sample Collection and Preparation

Two hadal snailfishes were collected from the Mariana Trench (141°56.8663’E, 10°59.2497’N; sample IDs: MT1 and MT2) at a depth of 7,034 m on 19th and 28th of July 2016 by the research ship “Tan Suo Yi Hao,” and one was obtained from the Yap Trench (138°32.3808’E, 9°43.6578’N; sample ID: YT1) at a depth of 7,884 m on 23rd of February 2017 also by “Tan Suo Yi Hao.” The locations for sampling are mapped in Supplementary Figure S1. The hadal snailfishes were trapped with baits using a sampler installed on a lander. The bait was wrapped with a high density sack to avoid direct ingestion. All of the hadal snailfishes were classified as *Pseudoliparis swirei* based on COI and morphology (Gerringer et al., 2017a). For comparison, two shallow-water snailfishes *Liparis tanakae* were collected from the southern central Yellow Sea by trawling at a depth of 20 m in November 2016. Specimens were dissected immediately once captured on board. To compare the microbial community structure in different gut segments, the guts were isolated and divided into three segments including front (Fgut), middle (Mgut), and hind (Hgut) segments, accordingly. Samples for molecular studies were stored at −80°C until use. Total DNA was extracted using PowerSoil DNA Isolation Kit (QIAGEN, United States). The quality and quantity of the extracted DNA were checked by gel electrophoresis and Qubit 2.0 Fluorometer (Life Technologies, United States), respectively.

### Cloning and Classification of 16S rRNA Gene Sequences

The full-length 16S rRNA genes of the microbes from snailfish guts were amplified with a pair of universal primers (27F: 5′-AGAGTTTGATC[C/A]TGGCTCAG-3′; 1492R: 5′-TACGG[C/T]TACCTTGTTACGAC-3′) (Wang et al., 2016). PCR amplification was performed in a 50 μl reaction containing 5 ng of bacterial DNA as template, 10 μl of 5 × PCR buffer, 4 μl of 10 mM dNTPs, 0.5 μl of Prime STAR HS DNA Polymerase (TaKaRa, Japan), 32.5 μl of ddH_2_O, and 2 μl of 10 μM each primer. The amplification program consisted of an initial denaturation step at 98°C for 10 s, followed by 25 cycles of 98°C for 10 s, 55°C for 15 s, and 72°C for 90 s, with a final extension of 10 min at 72°C. A no-template control was used to check for possible contaminations. The PCR products were separated by gel electrophoresis, and then the bands with expected size (∼1,500 bp) were purified with a QIAquick purification kit (Qiagen, Germany). After ligations into a vector pMD18-T (TaKaRa, Japan), the products were transformed into JM109 competent cells (TaKaRa, Japan). Thirty positive clones were randomly collected for each sample and sequenced using a Sanger sequencer (ABI 3730, United States). The chimera sequences were removed according to the previous study (Haas et al., 2011). Taxonomic assignment was based on the Silva online tools with default settings^1^. The classified sequences were further checked by searching against the NCBI database.

### Hybrid Sequencing, Metagenomic Assembly, Binning, and Annotation

A total of 200 ng of genomic DNA from the MT1 Hgut was employed for a paired-end library with insert size of 550 bp using TruSeq DNA Sample Prep Kit (Illumina, United States). The above library was sequenced twice on an Illumina MiSeq platform using V3 Miseq sequencing kits (2 × 300 bp) (Illumina, United States) to ensure sufficient data; 43.1 million 300 bp paired-end metagenomic reads were generated. Raw reads were processed with the NGS QC Toolkit version 2.3.3 (Patel and Jain, 2012) to remove short (<70 bp) and low quality (quality score <20) reads, followed by trimming adaptor sequences. Finally, 38.3 million clean reads were generated. Nanopore libraries were generated using 1D^2 sequencing kit (Oxford Nanopore, United Kingdom) with 1.5 μg of genomic DNA, and then sequenced using the Nanopore MinION system. 3.6 million long Nanopore reads (>4kb) were generated. Metagenomic assembly of the Illumina paired-end reads and Nanopore reads was performed using SPAdes 3.11 (Bankevich et al., 2012), with a maximum k-mer length of 127. The *Mycoplasma* draft genome was binned through separation based on different coverage levels and the G + C contents of the metagenomic contigs. The coverage levels of the assembled contigs were calculated using the idxstats module of SAMtools version 0.1.191 (Li et al., 2009). The binning process was visualized using RStudio^2^ according to a previous study (Albertsen et al., 2013). The coding sequences (CDSs) were predicted using Prodigal version 2.60 (Land et al., 2010), and configured with -g 4 and -p meta. The tRNA genes in the genome were identified using tRNAscan 1.3.1 (Lowe and Eddy, 1997). The quality and completeness of the draft genome were determined using CheckM (version 1.0.5) with the lineage workflow (Parks et al., 2015).

The annotation of deduced proteins was performed using BLASTp against four databases including NCBI Nr, KEGG (Ogata et al., 2000), Pfam (Finn et al., 2014), and COG (Sverdlov et al., 2003) with a maximum e-value cutoff of 1e-04. OrthoFinder 2.2.3 (Emms and Kelly, 2015) was used to further examine the shared and genome-specific genes among different bacteria with the default settings. The CRISPR region was identified using the CRISPRFinder online tool (Grissa et al., 2007).

### Reconstruction of Phylogenetic Trees

The 16S rRNA sequences used for the phylogenetic trees were obtained from NCBI and EzTaxon websites. Eight sequences from genus *Acholeplasma*, 22 from Entomoplasmatales, six from *Hepatoplasma*, nine from *Ureaplasma*, and 25 from *Mycoplasma* were selected for the phylogenetic tree with five *Bacillus* sequences used as the outgroup. The accession numbers are shown following the species names. Alignment was performed using MUSCLE3.5 with manual adjustments (Edgar, 2004), and a maximum-likelihood (ML) phylogenetic tree with 1,000 bootstraps was subsequently constructed using raxmlGUI (Silvestro and Michalak, 2012) with a GTRGAMMA model. The 16S rRNA sequences of mycoplasmas from different *P. swirei* individuals were also used to analyze the phylogenetic relationships with the same method. In addition, 54 Tenericutes genomes (including the current *Mycoplasma* genome) belonging to different taxonomic groups were pooled. Conserved single-copy genes (CSCGs) were identified by searching the CDSs against the Pfam database using Hmmsearch 3.0 with the default settings (Finn et al., 2014). A phylogenomic analysis was conducted using a total of 20 CSCGs (Supplementary Table S1) from a set of 31 genes previously defined for bacterial phylogenomic inference (Wu and Eisen, 2008). These 20 CSCGs were shared by all of the 54 genomes. Each of the 20 CSCGs was individually aligned with MUSCLE 3.5. The resulting aligned files were concatenated using a custom Perl script, and the resulting supermatrix was processed to estimate ML phylogeny with raxmlGUI with 1,000 bootstraps using PROTGAMMALG model, according to the previous study (He et al., 2017).

### Multilocus Gene Sequencing

The bacterial housekeeping genes (*atpA*, *recA*, and *gyrB*) were selected (Polzin et al., 2019) for PCR amplifications with a set of custom primers (Supplementary Table S2). DNA extracted from the guts was used as template. PCR reactions consisted of 50 μl of 1 μl DNA, 10 μl of 5 × PCR buffer, 4 μl of 10 mM dNTPs, 0.5 μl of Prime STAR HS DNA Polymerase (TaKaRa, Japan), 32.5 μl of ddH_2_O, and 2 μl of 10 μM each primer. The following program was applied: 98°C for 10 s; 30 cycles of 98°C for 10 s, annealing for 15 s, 72°C for 2 min, and a final extension of 10 min at 72°C. Annealing temperature was specific to each primer set (Supplementary Table S2). The PCR products were observed on 1% agarose gel electrophoresis. The amplicons with a correct size were sequenced using a Sanger sequencer (ABI 3730, United States).

## Results

### Gut Microbiota of the Snailfishes

**FIGURE 1:**
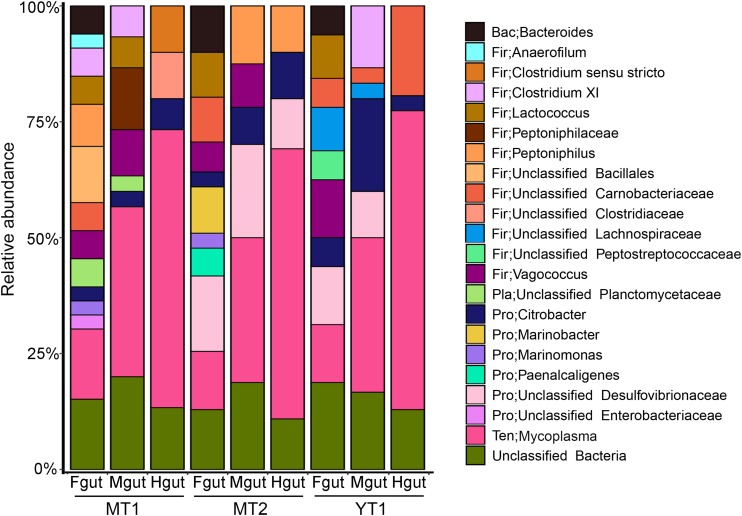
Bacterial communities in the hadal snailfish gut. A total of 270 clones for 16S rRNA sequences were randomly collected and sequenced. The Silva database was used as a reference for classification with default settings. Fgut: front segment of the gut; Mgut: middle segment of the gut; Hgut: hind segment of the gut; MT: snailfishes from the Mariana Trench; YT: snailfish from the Yap Trench.

Gut microbial communities in snailfishes were revealed by full-length 16S rRNA sequences (∼1,500 bp). A total of 270 clones from three *P. swirei* individuals were examined and classified into five phyla including Proteobacteria, Firmicutes, Tenericutes, Bacteroidetes, Planctomycetes, and unclassified one. In *P. swirei* Fguts, Firmicutes ranked the first with relative abundance of 35.0 ± 9.4%. For the second and third top phyla, Proteobacteria and Tenericutes occupied 21.7 ± 12.1% and 16.0 ± 2.9% of the microbial communities, respectively. However, *P. swirei* Mguts and Hguts were dominated by Tenericutes with relative abundances of 31.2–64.5% (Figure 1). The second and third top phyla were Firmicutes (18.8–29.2%) and Proteobacteria (6.7–21.6%). Moreover, *Mycoplasma* was the only genus from the Tenericutes phylum. The 16S rRNA sequences of mycoplasmas from different gut segments or different *P. swirei* individuals were nearly identical (>99.9% similarity). The relationship between different individuals was confirmed by phylogenetic analysis (Supplementary Figure S2). However, the gut microbiota of the two shallow-water snailfishes, *L. tanakae*, was dominated by the genera *Clostridium* and *Psychrobacter*, while *Mycoplasma* was absent (Supplementary Figure S3).

### Genome Sequence of *Mycoplasma*

Based on the analysis of gut microbiota, *Mycoplasma* was the dominant microbe in *P. swirei* guts, especially in Mguts and Hguts. To obtain more genomic information about the dominant *Mycoplasma*, metagenomic sequencing was performed. After optimized pipelines for assembly, the sequencing coverage for the assembled contigs, GC content, and taxonomic affiliation of the CSCGs derived from the contigs were analyzed in the RStudio platform with the libraries of vegan, plyr, RColorBrewer, and alphahull to classify these contigs into different genomes. Then, a draft genome of 795,917 bp for the dominant *Mycoplasma* was achieved at the coverage level of approximately 20-fold, with an extremely low G + C content of 23.5% (Table 1 and Supplementary Figure S4). The *Mycoplasma* of snailfish was 97.8% complete and included three contigs, 750 predicted protein-coding genes, 95 CSCGs, 30 tRNA genes, and a complete set of tRNA genes for 20 amino acids. To further investigate whether the dominant *Mycoplasma* species from *P. swirei* guts were polyclonal or monoclonal, the housekeeping genes *atpA*, *recA*, and *gyrB* were amplified and sequenced from the three *P. swirei* Hguts. The three housekeeping gene sequences revealed no variation (100% identity) across all the Hguts. These results indicated that a monoclonal *Mycoplasma* strain might exist in the *P. swirei* guts.

**TABLE 1:** Genome features and KEGG pathways of deep-sea symbiotic Tenericutes.

**Genome features and KEGG pathways**	**CML**	**CSH**	**MB**
Genome size (bp)	795,917	424,539	785,028
Contig number	3	2	5
GC content (%)	23.5	29.6	26.5
CDS number	750	347	678
tRNA genes	30	32	33
rRNA genes	3	2	3
Coding density (%)	86.8	90.2	91.8
Conserved single-copy genes	95	98	101
CheckM completeness (%)	97.8	/	/
CheckM contamination (%)	0.0	/	/
ko00220 Arginine biosynthesis	3	3	3
ko00740 Riboflavin metabolism	7	1	2
ko02010 ABC transporters	12	6	10
ko02060 Phosphotransferase system	1	0	12
References	This study	He et al., 2017	Wang et al., 2016

*CML: the genome of “*Ca*. Mycoplasma liparidae” (PRJNA497967); CSH: “*Ca.* Spiroplasma holothuricola” (CP022928/9), a hadal holothurian symbiont; MB: mycoplasma BG1 (LSMX00000000), isolated from a deep-sea isopod.*

Compared with the other two genomes of dominant Tenericutes from the deep-sea cucumber and the isopod *Bathynomus* sp. (Table 1), *Mycoplasma* of snailfish was larger in size, while coding density was slightly sparse. In terms of metabolic pathways, *Mycoplasma* of snailfish contained more genes encoding riboflavin metabolism and ABC transporters, but had fewer phosphotransferase transport systems than mycoplasma BG1.

### Phylogenetic Analysis

**FIGURE 2:**
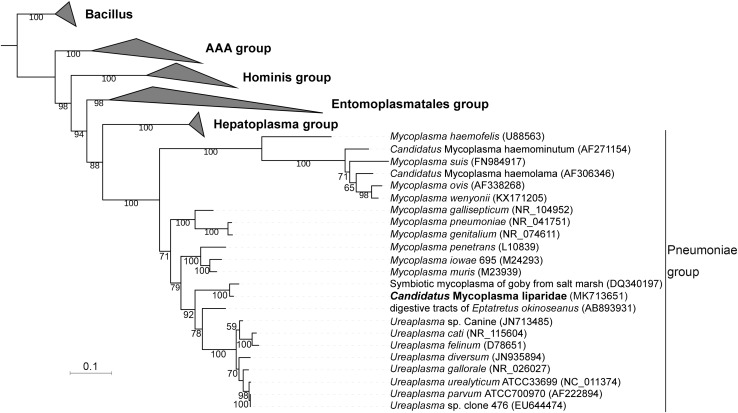
Phylogenetic position of “*Ca.* Mycoplasma liparidae.” The 16S rRNA sequence of “*Ca.* Mycoplasma liparidae” was aligned with those of 69 Tenericutes to reconstruct a maximum-likelihood tree with 1,000 replications. The bootstrap values are indicated on the branches of the tree. If a sequence is not associated with a species name, the host and source are indicated. The species investigated in this study is highlighted.

A total of 70 sequences from five groups in Tenericutes were used to construct a phylogenetic tree. The current *Mycoplasma* was grouped within the pneumoniae group and approximated to a symbiotic *Mycoplasma* from the goby gut (97% identity for 16S rRNA sequences), which was supported by a bootstrap value of 100 (Figure 2). Compared with *Mycoplasma muris*, the snailfish *Mycoplasma* was more distantly related to members of *Ureaplasma*. The high genetic relatedness of snailfish *Mycoplasma* with other *Mycoplasma* species was further substantiated by a phylogenomic analysis using 53 additional genomes from different taxonomic groups in Tenericutes (Supplementary Figure S5). Phylogenetic analysis revealed that snailfish *Mycoplasma* represents a novel lineage between *Mycoplasma* group and *Ureaplasma* group. Thus, the species named “*Candidatus* Mycoplasma liparidae” is proposed for this bacterium (hereafter, genome of “*Candidatus* Mycoplasma liparidae”; CML).

To further understand the relationship between “*Ca.* Mycoplasma liparidae” and other Tenericutes, we selected 29 Tenericutes genomes to calculate the average nucleotide identity (ANI). The ANI value ranged from 60.9 to 65.5% with a standard deviation of 0.79% (Supplementary Table S3), showing that the closest known relative of “*Ca.* Mycoplasma liparidae” was *Mycoplasma iowae*.

### Metabolic Features

**FIGURE 3:**
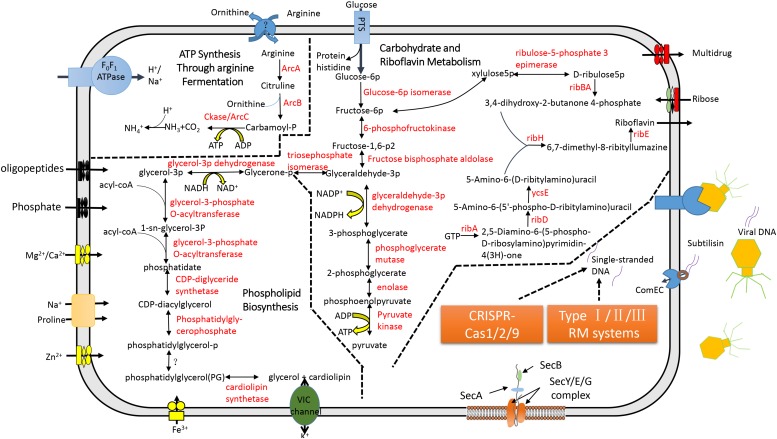
Metabolic pathways and the CRISPR-Cas system in “*Ca.* Mycoplasma liparidae.” The primary metabolic pathways in “*Ca.* Mycoplasma liparidae” were predicted in an integrated manner. Metabolic products are shown in black, and enzymes or proteins are shown in red. Question marks denote transporters or enzymes that were not identified.

In the CML, a single phosphotransferase system (PTS) was identified (Figure 3). But, CML did not possess genes involved in pyruvate degradation and tricarboxylic acid cycle (Figure 3). “*Ca.* Mycoplasma liparidae” may produce ATP through the fermentation of arginine (arginolysis). All the genes encoding the enzymes that took part in arginolysis via the arginine deiminase system (ADI pathway) were identified in the CML (Figure 3). The arginine deiminase gene (*arcA*) in the CML shared its highest identity (70%) to *Mycoplasma moatsii*. Carbamoyl-P, a product of citrulline degradation, is likely catabolized by carbamate kinase (Ckase) to generate ATP, ammonia, and CO_2_. However, only one gene (*gpmI*) was involved in glycine, serine, and threonine metabolism; six genes (*metK*, four *dnmT* and *mtnN*) were involved in methionine metabolism, but the key genes (*metA*, *metB*, *metC*, and *metE*) of methionine biosynthesis were lost; one gene (*dapF*) was responsible for lysine biosynthesis; four genes (four *aguA*) were involved in proline metabolism; and one gene (*katA*) was involved in tryptophan metabolism. No gene was related to the metabolism of the remaining amino acids. Amino acid biosynthetic genes were almost entirely eliminated, suggesting that “*Ca.* Mycoplasma liparidae” may obtain amino acids from other sources, such as the host.

Notably, CML was found to harbor all of the key genes involved in the biosynthesis of riboflavin (vitamin B_2_), including *ribA*, *ribD*, *ribZ* (*ycsE*), *ribBA*, *ribH*, and *ribE* (Figure 3). A comparison of the gene order at the corresponding genome region in the six genomes showed that *ribD*, *ribE*, *ribBA*, and *ribH* usually clustered together and were conservatively present (Supplementary Figure S6A). Riboflavin synthase (*ribE*), a critical enzyme, catalyzes the final step in the riboflavin biosynthetic pathway to yield riboflavin. The riboflavin synthase in “*Ca.* Mycoplasma liparidae” has not been reported in Mollicutes. The most similar homolog was from a *Clostridium* sp. CAG (49% identity at amino acid level). The phylogenetic tree revealed that CML-derived riboflavin synthase branched together with that in *Clostridium* sp. CAG (Supplementary Figure S6B). To examine the presence of a binding site of the substrate for riboflavin synthase in “*Ca.* Mycoplasma liparidae,” the alignment of amino acid sequences was performed with three well-characterized homologs to show the conserved sites and motifs (Supplementary Figure S7).

### Absence of Virulence Factors

**FIGURE 4:**
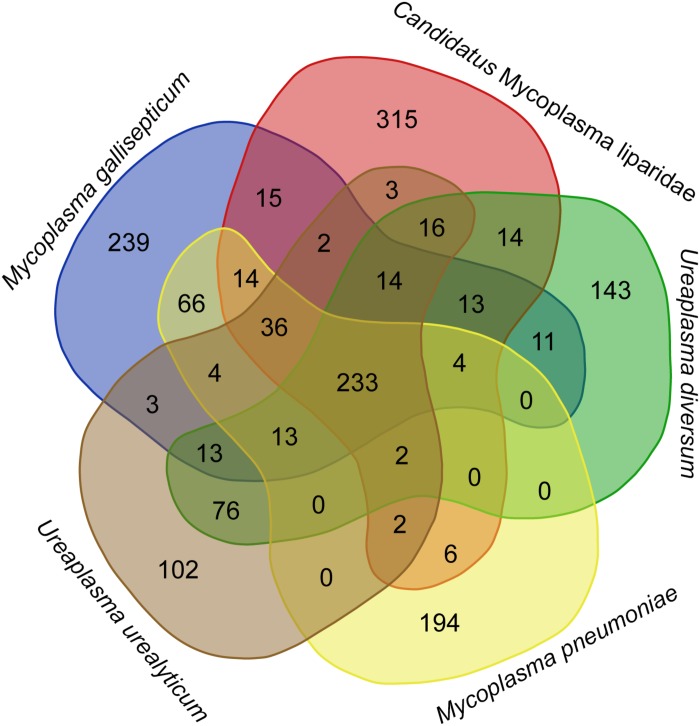
Diagram showing shared and species-specific gene clusters. Gene content was compared among “*Ca.* Mycoplasma liparidae” (PRJNA497967), *U. urealyticum* (NC_011374), *U. diversum* (CP009770), *M. gallisepticum* (NC_004829), and *M. pneumoniae* (NC_000912). Orthogroup analysis was used to show the common genes between these bacterial genomes.

The CML lacks a *glpD* gene, which is the key gene for H_2_O_2_ production via a glycerol catabolic pathway, but encodes a catalase (54% identity to the homolog of *M. iowae*) for H_2_O_2_ degradation. To gain further insight into the genetic disparity between “*Ca.* Mycoplasma liparidae” and pathogenic bacteria, we compared the gene content of “*Ca.* Mycoplasma liparidae” with that of four pathogens, namely, *Ureaplasma urealyticum*, *Ureaplasma diversum*, *Mycoplasma gallisepticum*, and *Mycoplasma pneumoniae*, all belonging to the pneumoniae group. Their core genome comprised 233 genes (Figure 4). As expected, the functions of these common genes were concentrated in translation, ribosomal structure, and biogenesis. Nevertheless, the genomes of the four pathogens have some particular genes encoding multiple-banded antigen, IgA protease, urease, ADP-ribosylating, and other virulence factors. All of these virulence genes were absent from CML (Supplementary Table S4). Intriguingly, CML harbored 315 unique genes, of which only 69 genes had KEGG orthology. A total of 171 genes corresponded to proteins with an unknown function.

### Immune Protection

**FIGURE 5:**
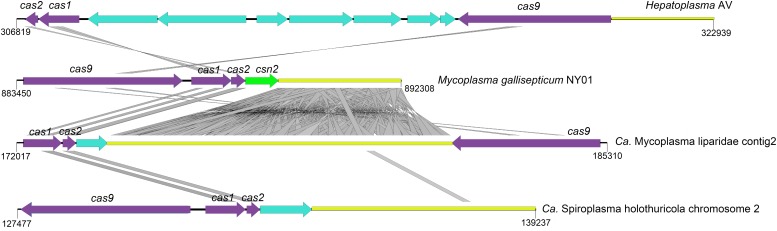
Structures of CRISPR-Cas systems in Tenericutes. The loci for *cas*, *csn*, and CRISPR are shown in purple, green, and yellow, respectively. Genes unrelated to the CRISPR-Cas system are colored cyan. tBLASTx comparisons were performed with an e-value of 10^–9^, and homologous regions are indicated with gray frames. The reverse orientation of *cas* occurred in “*Ca.* Mycoplasma liparidae” (hadal snailfish symbiont), “*Ca*. Spiroplasma holothuricola” (hadal holothurian symbiont), and “*Candidatus* Hepatoplasma crinochetorum” (terrestrial isopods symbiont).

Here, a CRISPR-Cas system consisting of three *cas* genes (*cas1/cas2/cas9*) and 118 spacers were detected in CML. Homologous genes with the highest identity were found in the genomes of *M. moatsii* (48% for *cas1*; 44% for *cas2*) and *Mycoplasma alvi* (28% for *cas9*). However, an unusual organization of CRISPR-Cas genes was displayed in CML compared to that of *M. gallisepticum*. The *cas9* gene was located downstream of the CRISPR locus and was in a reverse orientation (Figure 5).

To identify the matching viruses, the sequences of CRISPR spacers were searched against the virus collection in the NCBI database (NCBI taxid 10,239), and the following viruses and phages were identified: 0.85% of the spacers (one spacer) matched *Acanthamoeba castellanii* mimivirus (100% identity in nucleotide sequence), 0.85% of the spacers matched *Chrysochromulina ericina* virus (93% identity), and 0.85% of the spacers matched Megavirus (100% identity); 2.54% of the spacers matched *Lactococcus* phage (92% identity), 4.23% of the spacers matched *Bacillus* phage (92% identity), 2.54% of the spacers matched *Vibrio* phage (95% identity), and 0.85% of the spacers matched *Hydrogenobaculum* phage (92% identity) (Supplementary Table S5).

## Discussion

In this study, three *P. swirei* individuals from two trenches displayed the same taxa, “*Ca.* Mycoplasma liparidae,” in the guts, which were indicated to be the same strain by analyses of the 16S rRNA gene and three housekeeping genes. The presence of several similar bacterial taxa in the gut-associated microbiota of the same or multiple fish species from different geographical locations indicated that these ribotypes might be important contributors to gut functions (Roeselers et al., 2011; Ghanbari et al., 2015), referred to as the “core gut microbiota” (Rawls et al., 2004; Kessel et al., 2011; Wong et al., 2013). Such a gut symbiosis system was restricted to hadal snailfish species, as we did not detect a similar gut microbiota in shallow-sea snailfish. In “*Ca.* Mycoplasma liparidae,” glucose can be transported from outside and degraded into pyruvate via the Embden-Meyerhof-Parnas pathway (Robillard and Broos, 1999). However, due to absence of oxidative phosphorylation systems, arginolysis may be an alternative process for the generation of ATP (Vander et al., 1984) in “*Ca.* Mycoplasma liparidae.”

The genome of the hadal snailfish *P. swirei* revealed no genes associated with riboflavin biosynthesis (Wang et al., 2019), but a complete set of genes involved in riboflavin biosynthesis was present in the genome of “*Ca.* Mycoplasma liparidae,” indicating that *P. swirei* may largely depend on “*Ca.* Mycoplasma liparidae” for the supply of riboflavin when it was insufficient in foods. Riboflavin biosynthesis occurs in many bacteria, such as *Bacillus subtilis* and *Escherichia coli* (Leblanc et al., 2013), but is quite unusual in Tenericutes. To the best of our knowledge, this is the first report to show that *Mycoplasma* can synthesize riboflavin. Riboflavin synthase is the key enzyme for riboflavin biosynthesis and has no homolog in other Tenericutes but shares 49% identity with *Clostridium*, suggesting the occurrence of horizontal gene transfer. As expected, “*Ca.* Mycoplasma liparidae” and *Clostridium* sp. CAG were placed into one clade by phylogenetic analysis using riboflavin synthases. In addition, the alignment of amino acid sequences with three well-characterized homologs (Eberhardt et al., 1996) indicates the presence of binding sites in riboflavin synthase of CML. Instead, virulence factors were absent in the CML, but common to other pathogens (Glass et al., 2000; Kannan and Baseman, 2006; Szczepanek et al., 2010; Indikova et al., 2013; Kokkayil and Dhawan, 2015) and genes involved in the generation of H_2_O_2_, which is a major mediator for *Mycoplasma* pathogens to cause cellular damage (Pritchard et al., 2014), were also absent in the CML. All these results indicated that “*Ca.* Mycoplasma liparidae” co-evolved with host and other bacteria during symbiosis. Phylogenetic trees using both the 16S rRNA genes and genomes showed that “*Ca.* Mycoplasma liparidae” was almost equidistant from *Mycoplasma* and *Ureaplasma*, which is not a novel phenomenon. It has been reported that symbiotic *Mycoplasma* from the digestive tracts of scorpions represent a novel *Mycoplasma* group according to 16S rRNA sequence analysis (Elmnasri et al., 2018); symbionts of termites have been classified as a new lineage within the *Mycoplasma* genus (Hongoh et al., 2003). Not limited to Tenericutes, intracellular symbionts residing in the bacteriomes of leafhoppers fell into a well-defined clade within Proteobacteria (Moran et al., 2003). Similarly, the *Spiroplasma* symbiont from the gut of the hadal sea cucumber was distantly related to known species (He et al., 2017), probably due to genetic drift during a long-term symbiosis.

Deep-sea waters and sediments are replete with viruses (Hara et al., 1996; Danovaro et al., 2008). The development of an efficient immune system to address this situation is the key to survival. CRISPR-Cas system confers adaptive immunity for prokaryotes against mobile elements and phage infections (Deveau et al., 2010; Barrangou and Marraffini, 2014). In this study, the CRISPR system encoded by CML may prevent viral infections to protect snailfishes in three aspects: (i) against phages to maintain normal gut microbiota; (ii) against viruses infecting the host; and (iii) against viruses infecting the gut parasite that potentially regulates homeostasis of the host. It is possible that the one end of “*Ca.* Mycoplasma liparidae” may be exposed to the outside of the gut epithelial cell, suggesting its ability to capture and degrade some phages in the snailfish gut, and the other end of “*Ca.* Mycoplasma liparidae” enters the inside of the gut cell, underlying it could probably destroy the viruses infecting the host. “*Ca.* Mycoplasma liparidae,” which may be partially embedded in the gut wall, resembles the human pathogen *Mycoplasma genitalium*, which infects lung cells (Jensen et al., 1994). At present, several CRISPR-Cas subtypes have been identified, each with a specific set of *cas* genes (Makarova et al., 2015). The *cas9* gene in the CML is a type II CRISPR-Cas subtype, usually composed of *cas9*, *cas1*, *cas2*, and *cas4* (for type IIB) or *csn2* (for type IIA) genes or only three *cas* genes (for type IIC), all located in a single transcriptional unit directly upstream of the CRISPR locus (Zhang et al., 2013; Makarova et al., 2015). Interestingly, the *cas9* gene was located downstream of the CRISPR locus and was in a reverse orientation in the “*Ca.* Mycoplasma liparidae” genome. The reverse orientation of the *cas* gene has also been observed in symbionts of hadal holothurian and terrestrial isopods. In addition, all of the above three symbionts lacked the *csn2* gene. This type of CRISPR-Cas system with *cas* gene rearrangements was thought to be largely inefficient to against further infections (Leclercq et al., 2014). However, type IIC systems that stimulate immunity without the *csn2* gene have been proposed (Zhang et al., 2013; Chylinski et al., 2014). A recent study reported a new subtype CRISPR-Cas system lacking the *csn2* gene in an uncultivated archaeal strain (Burstein et al., 2017), in which the locus organization was similar to that of “*Ca.* Mycoplasma liparidae.” Our findings indicated that the *cas* gene rearrangements may point toward the evolution of a new CRISPR-Cas subtype.

## Data Availability Statement

Sequencing data has been deposited in the NCBI GenBank. The CML has been deposited at GenBank under the BioProject accession number: PRJNA497967. The 16S rRNA gene sequence is accessible in GenBank under the accession number: MK713651.

## Ethics Statement

Mariana and Yap hadal snailfish specimens were trapped using deep-sea landers carried by the research ship “Tan Suo Yi Hao.” The captured hadal snailfishes were dead once collected on deck. Wild shallow-water snailfishes were collected from a depth of 20 m in the southern central Yellow Sea by trawling. All of the snailfishes are neither endangered nor protected animals. Animal sampling was carried out in accordance with the animal experimentation guidelines and regulations of the Institute of Deep-Sea Science and Engineering. The protocol was approved by the Federated States of Micronesia, with permit numbers FM16-CN20162RS-01 and FM17-CN20162RS-01, respectively.

## Author Contributions

C-AL, YW, and L-SH conceived and designed the experiments. G-YY, AD, and J-MH performed the experiments and analyzed the data. C-AL and L-SH wrote the manuscript with input from all other members. YW and L-SH directed and supervised all of the research.

## Conflict of Interest

The authors declare that the research was conducted in the absence of any commercial or financial relationships that could be construed as a potential conflict of interest.

## Footnotes

1 https://www.arb-silva.de

2 https://www.rstudio.com
