# Supplementary material for: Genomic Characterization of a Novel Gut Symbiont From the Hadal Snailfish
Source: Front Microbiol. 2020 Jan 10;10:2978. doi: 10.3389/fmicb.2019.02978 (PMC6965317; doi:10.3389/fmicb.2019.02978)
Supplement: Supplementary file 1 [file Data_Sheet_1.pdf]

Genomic characterization of a novel gut symbiont from the hadal snailfish

Chun-Ang Lian,[1,2] Guo-Yong Yan,[1,2] Jiao-Mei Huang,[1,2] Antoine Danchin,[3] Yong Wang,[1,*] and Li-Sheng He[1,*]

[1] Institute of Deep-sea Science and Engineering, Chinese Academy of Sciences, Sanya, Hainan, China

[2] University of Chinese Academy of Sciences, Beijing, China

[3] Institute of Cardiometabolism and Nutrition, Hôpital de la Pitié-Salpêtrière, 47 boulevard de l'Hôpital, 75013 Paris, France

* Correspondence:

Li-Sheng He

he-lisheng@idsse.ac.cn

Tel.: +86-898-8838-0060

Fax: +86-898-8822-2506

and

Yong Wang

wangy@idsse.ac.cn

Keywords: hadal symbiosis; Tenericutes; CRISPR; snailfish; metagenome
Running title: Symbiotic *Mycoplasma* in hadal snailfish gut

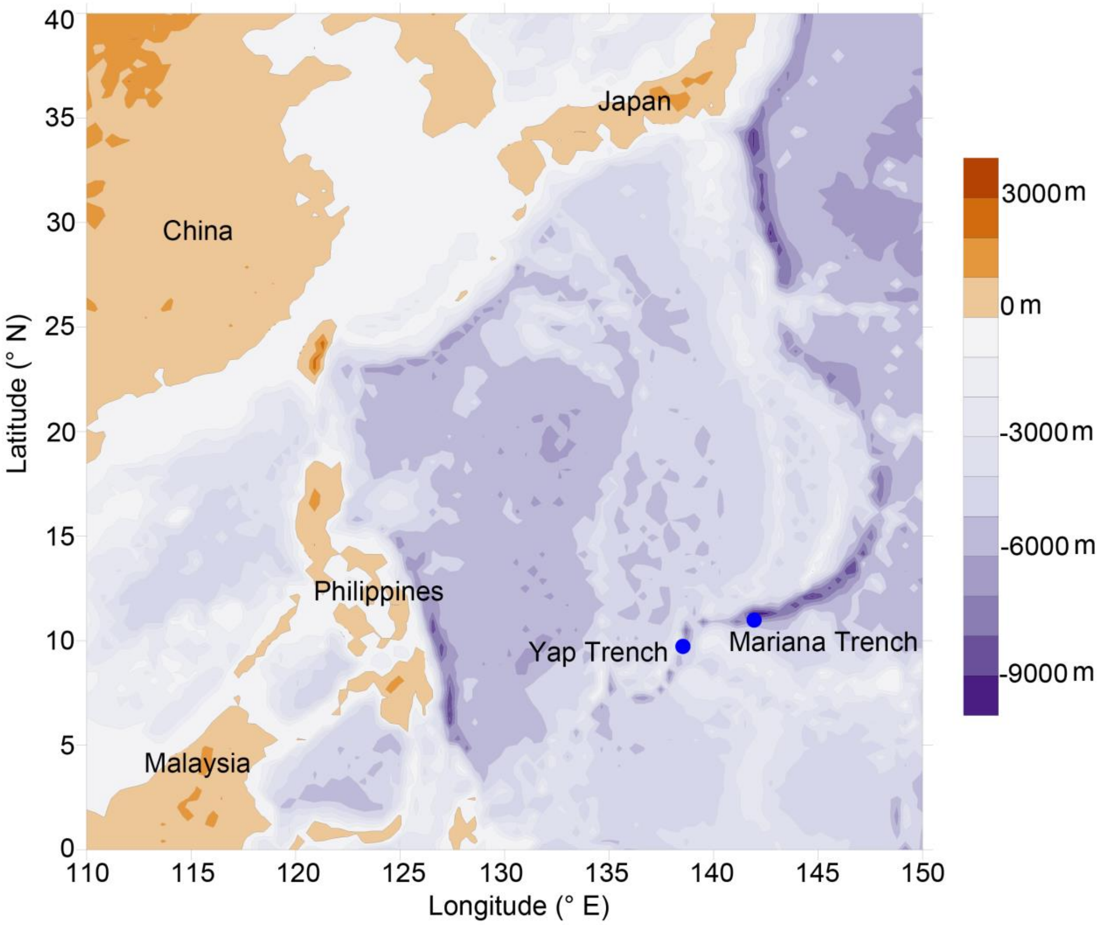

Figure S1. Sampling locations of hadal snailfishes

Hadal snailfishes were collected from the Mariana and Yap Trench. The sampling locations are marked with blue dots.

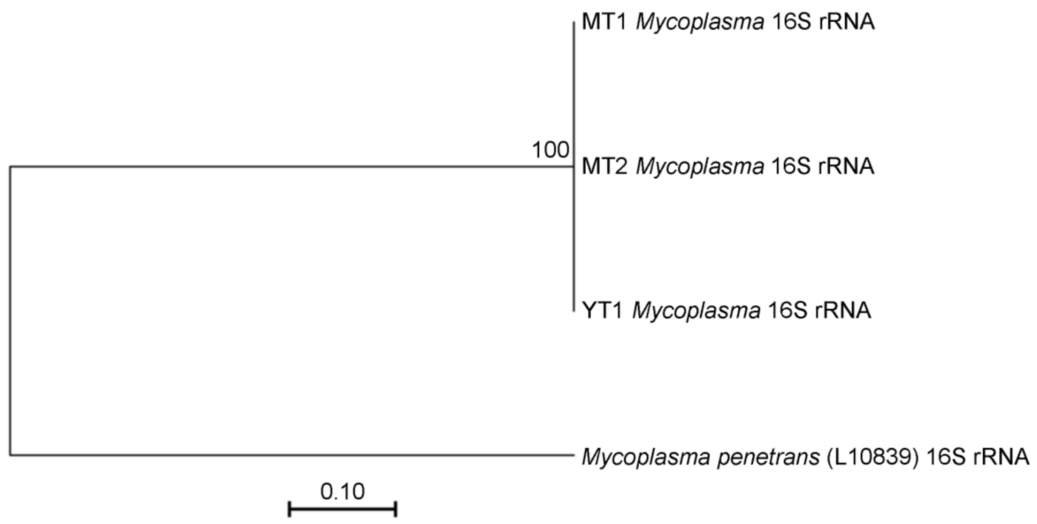

Figure S2. Phylogenetic relationships of 16S rRNA genes between different individual snailfish

The maximum-likelihood tree was constructed based on the nearly full-length 16S rRNA sequences (~1,500 bp). The bootstrap values were based on 1,000 permutations, and the 16S rRNA from *Mycoplasma penetrans* was used as the root.

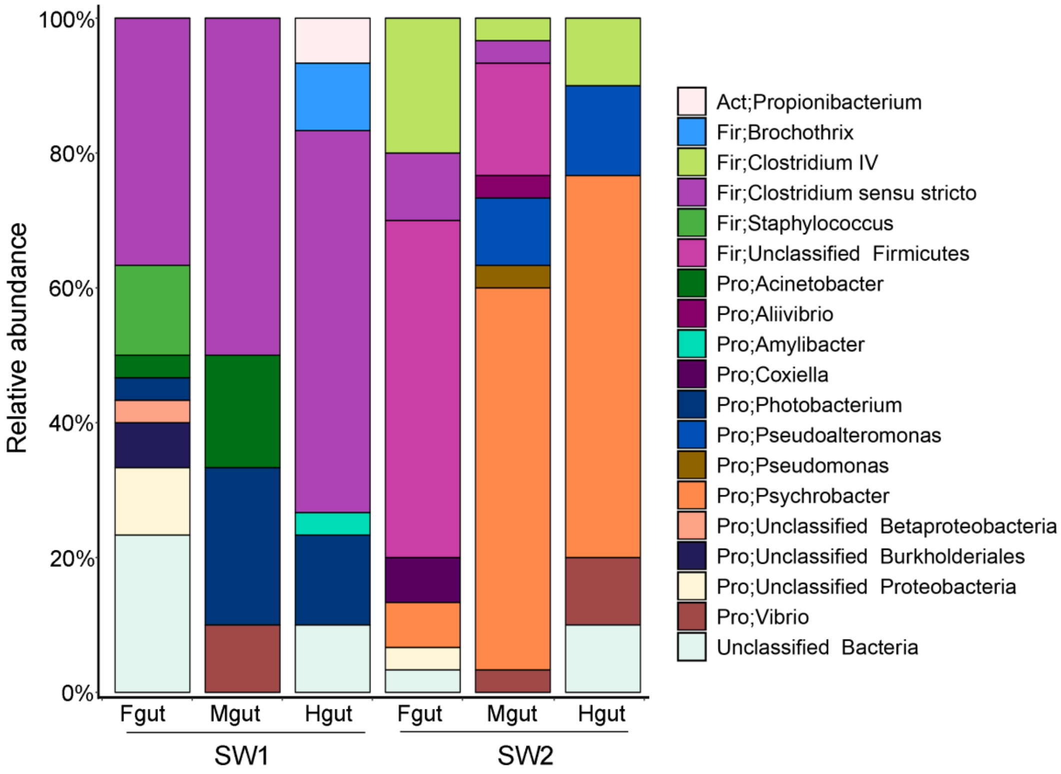

Figure S3. Bacterial community in the guts of shallow-water snailfishes

Fgut, Mgut and Hgut represent the front, middle, and hind segments of the gut, respectively. The 16S rRNA from the Fgut, Mgut and Hgut of the snailfish were amplified and cloned into T-vectors. A total of 180 positive clones were randomly collected and sequenced. The Silva database was used for classification with default settings. The classified sequences were checked again using the NCBI database.

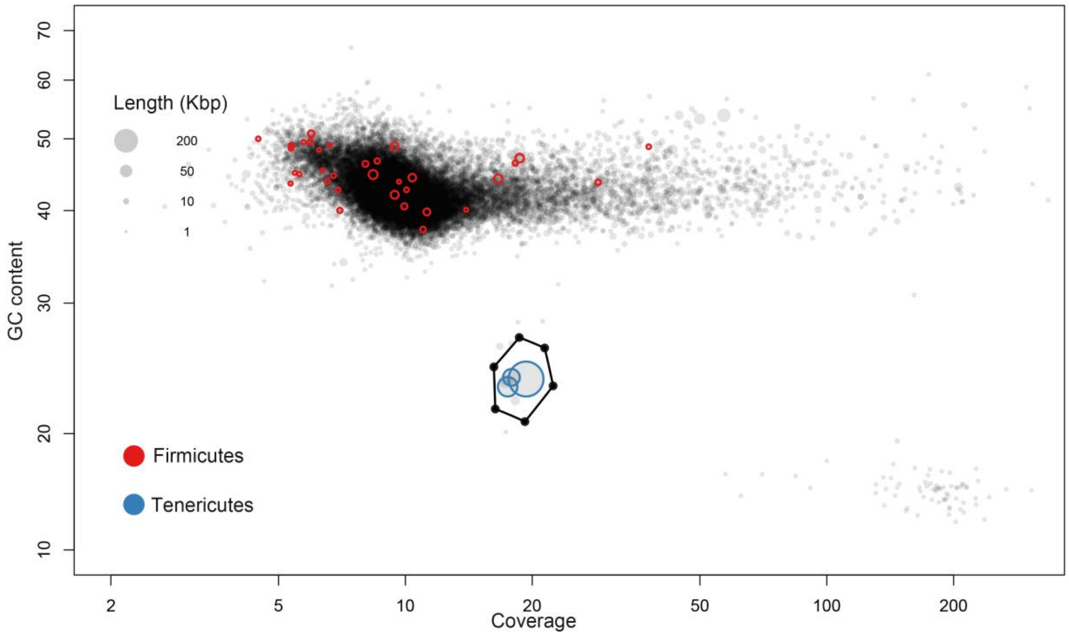

Figure S4. Binning of draft genomes

The genomic DNA from the hadal snailfish Hgut was extracted and then sequenced. The sequencing reads were assembled into contigs. The coverage levels of the contigs in terms of the metagenome were calculated, and the draft genomes were binned out.

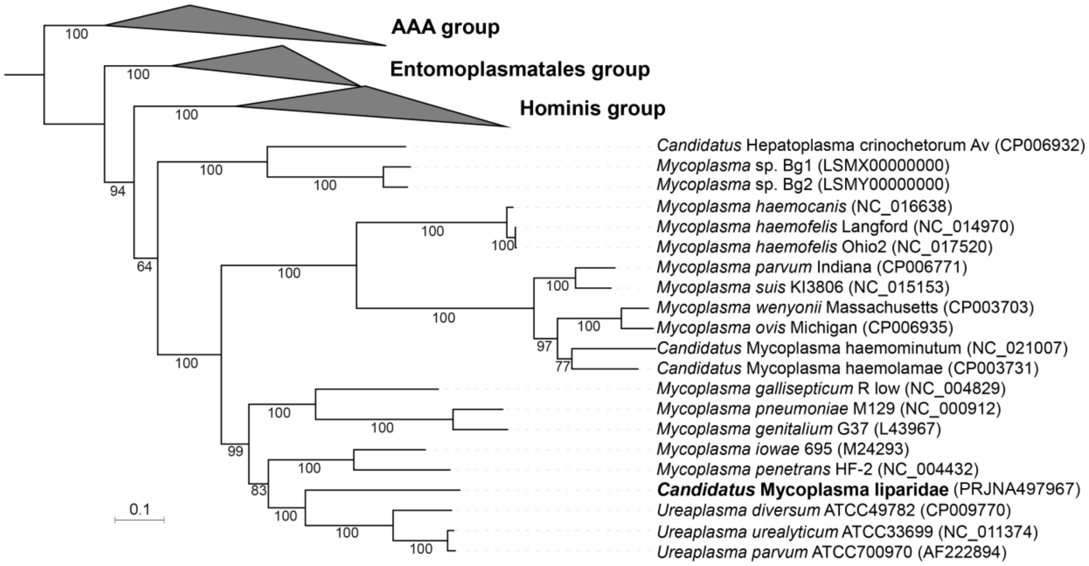

Figure S5. Phylogenomic tree constructed using conserved genes

A phylogenetic analysis using 53 additional genomes belonging to different taxonomic groups was conducted. A total of 20 CSCGs were extracted from all the genomes and individually aligned using MUSCLE3.5. Aligned files were concatenated to construct a maximum-likelihood tree.

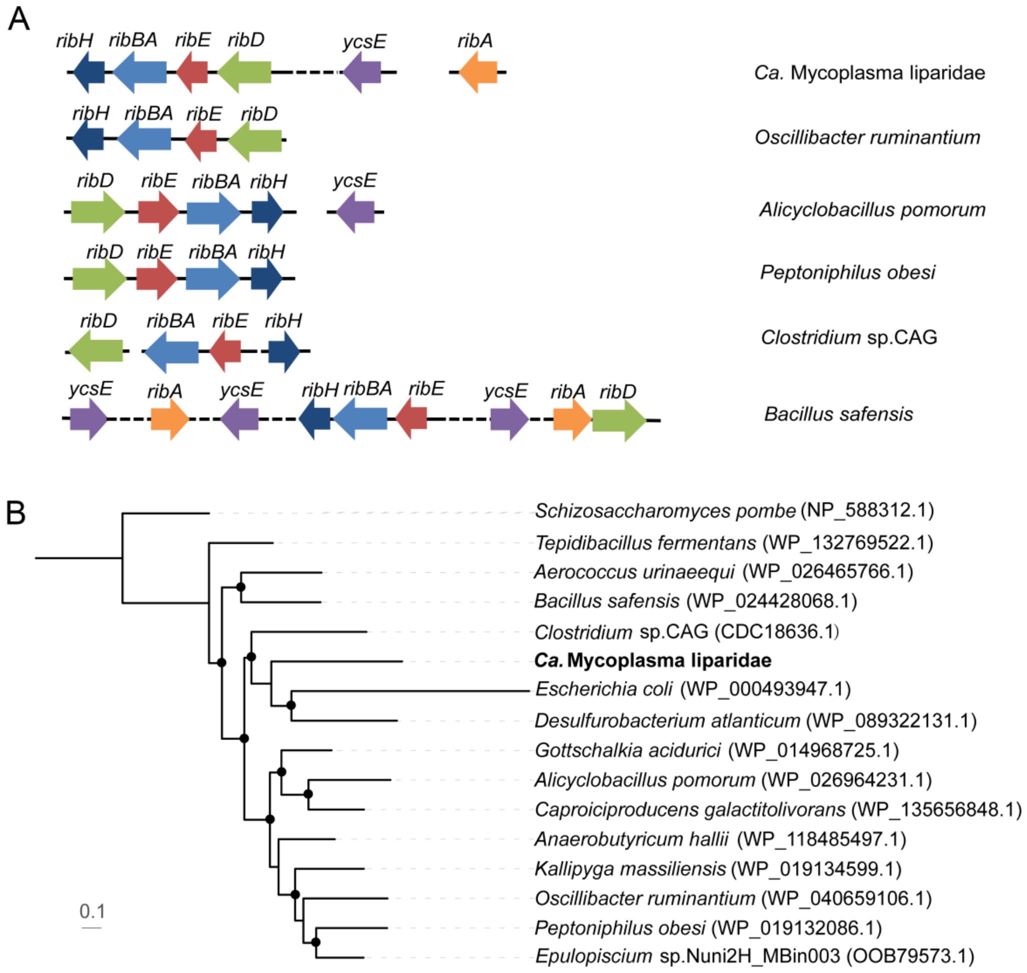

Figure S6. Phylogeny of riboflavin synthase and synteny of genes involved in riboflavin biosynthesis

The genes involved in the biosynthesis of riboflavin are shown above and labeled with the corresponding name. If the gene interval was 10 kb or more, it is indicated by a broken line (A). Riboflavin synthase from 16 genomes was collected to reconstruct a maximum-likelihood tree with 1,000 replicates. Nodes with bootstrap values >50% are marked with solid dots (B).

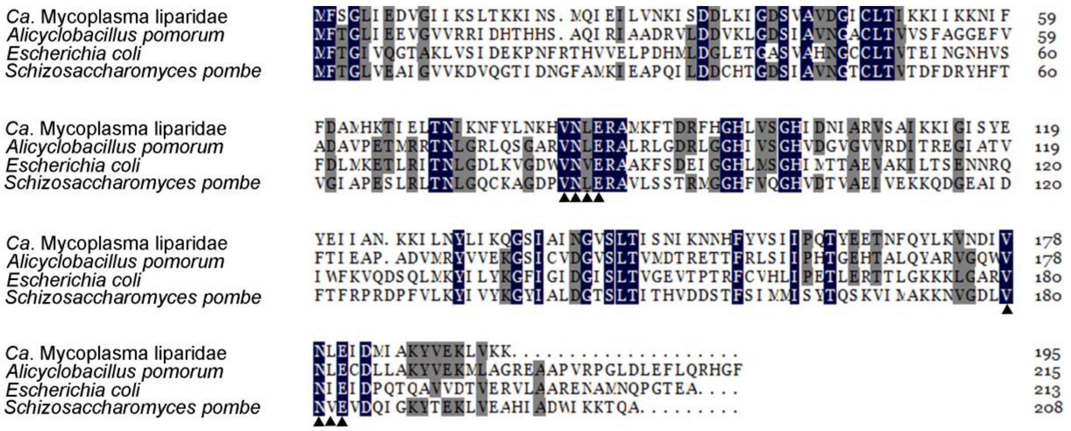

Figure S7. Alignment of riboflavin synthase sequences

The riboflavin synthase sequence from “*Ca.* Mycoplasma liparidae” was aligned with those of three homologues from *Alicyclobacillus pomorum*, *Escherichia coli* and *Schizosaccharomyces pombe*. The proposed binding site of the substrate is marked by a solid triangle.

Table S1 CSCGs were used to construct a phylogenomic tree

conserved single-copy genes（CSCGs）	pfam or TIGRFAM
dnaG	TIGR01391
frr	TIGR00496
nusA	TIGR01953
rplA	TIGR01169
rplB	TIGR01171
rplD	TIGR03953
rplE	TIGR01021
rplF	pfam00347
rplK	pfam00411
rplL	TIGR00855
rplM	TIGR01066
rplN	TIGR01066
rplP	TIGR01164
rpsB	TIGR01011
rpsC	TIGR01009
rpsE	TIGR01164
rpsI	pfam00380
rpsM	pfam00416
tsf	TIGR00116
smpB	TIGR00086

Table S2 Primers used for PCR amplification

Gene	Primer sequences (5'-3')	fragment (bp)	Annealing temperature (˚C)
atpA	forw.: GTTATTTCTTTAGGTGATGGT<br>rev.: ATAACTGACCATCTGTAATTG	937	45
recA	forw.: AACAAAATTAATGTTGATGC<br>rev.: TAAACGTAATGTTTCTTC	957	42
gyrB	forw.: GATTAATGATAAAAAAGATG<br>rev.: CATTCTTAGCAATAAATTCTT	1967	42

Table S3 Tenericutes species used in the ANI survey

Species name	ANI values
Mycoplasma iowae 695	65.49
Ureaplasma urealyticum serovar 10 ATCC 33699	65.34
Mycoplasma penetrans HF-2	65.24
Ureaplasma diversum ATCC49782	65.05
Ureaplasma parvum serovar 3 ATCC 700970	65.05
Mycoplasma gallisepticum R	64.66
Candidatus Mycoplasma haemominutum	64.54
Mycoplasma suis KI3806	64.43
Mycoplasma haemofelis str.Langford 1	64.3
Mycoplasma parvum str.Indiana	64.22
Mycoplasma ovis str.Michigan	63.98
Spiroplasma diminutum CUAS 1	63.97
Mycoplasma wenyonii str.Massachusetts	63.91
Candidatus Hepatoplasma crinochetorum	63.88
Candidatus Mycoplasma haemolamae str.Purdue	63.85
Mycoplasma haemocanis str.Illinois	63.82
Mycoplasma genitalium G37	63.67
Mycoplasma synoviae 53	63.64
Mycoplasma pulmonis	63.64
Mycoplasma bovis PG45	63.41
Mycoplasma hyorhinis str.HUB-1	63.31
Acholeplasma laidlawii	63.29
mycoplasma BG1	63.2
Strawberry lethal yellows phytoplasma (CPA) str.NZSb11	62.96
Candidatus Phytoplasma australiense	62.95
Onion yellows phytoplasma	62.24
Acholeplasma brassicae	61.76
Mycoplasma pneumoniae M129	61.58
Candidatus Phytoplasma solani 284/09	60.98

All the species have complete genome sequences in the NCBI database.

Table S4 Comparison of "*Ca.* Mycoplasma liparidae" with other pathogens

virulence factors	CML	UU	UD	MG	MP
Multiple Banded Antigen	N	Y	N	N	N
IgA protease	N	Y	N	N	N
Urease	N	Y	Y	N	N
phospholipases A and C	N	Y	N	N	N
GapA	N	N	N	Y	N
CrmA	N	N	N	Y	N
MslA	N	N	N	Y	N
ADP-ribosylating	N	N	N	N	Y

Y indicates present; N indicates absent. CML: "*Ca.* Mycoplasma liparidae" (BioProject accession number PRJNA497967); UU: *U. urealyticum* (NC_011374); UD: *U. diversum* (CP009770); MG: *M. gallisepticum* (NC_004829); MP: *M. pneumoniae* (NC_000912).

Table S5 Spacers matched viruses or phages

number of spacers	percentage (%)	matched viruses/phages	identity (%)
1	0.85	*Acanthamoeba castellanii* mimivirus	100
1	0.85	*Chrysochromulina ericina* virus	93
1	0.85	Megavirus	100
3	2.54	*Lactococcus* phage	92
5	4.23	*Bacillus* phage	92
3	2.54	*Vibrio* phage	95
1	0.85	*Hydrogenobaculum* phage	92
